# Effectiveness of a Small Private Online Course in Sexual Health Communication for Registered Nurses: A Randomized Controlled Trial

**DOI:** 10.1097/jnr.0000000000000751

**Published:** 2026-08-19

**Authors:** Hsien-Tsung CHANG, Jia-Ling TSAI, Chiu-Tzu LIN, Ding-Hau HUANG, Guang-Wu CHEN, Nien-Ting CHANG, Yu LEE, Jian Tao LEE

**Affiliations:** 1 Bachelor Program in Artificial Intelligence, Chang Gung University, Taoyuan, Taiwan; 2 Department of Computer Science and Information Engineering, Chang Gung University, Taoyuan, Taiwan; 3 Department of Physical Medicine and Rehabilitation, Chang Gung Memorial Hospital, Taoyuan, Taiwan; 4 School of Nursing, College of Medicine, Chang Gung University, Taoyuan, Taiwan; 5 Nursing Department, New Taipei City Municipal TuCheng Hospital, New Taipei City, Taiwan; 6 Department of Nursing, Chang Gung University of Science and Technology, Taoyuan, Taiwan; 7 Institute of Creative Design and Management, National Taipei University of Business, Taipei, Taiwan; 8 Nursing Department, Chang Gung Memorial Hospital, Taipei Branch, Taipei, Taiwan; 9 Ministry of Health and Welfare Shuang-Ho Hospital, New Taipei, Taiwan; 10 Nursing Department, Chang Gung Memorial Hospital, Linkou Branch, Taoyuan, Taiwan

**Keywords:** sexual health, communication, nursing education, health knowledge, attitudes, randomized controlled trial

## Abstract

**Background:**

Small Private Online Courses (SPOCs) apply a small-group, blended learning format to facilitate instructor–learner interactions. SPOCs are ideal for time-limited learners in clinical settings.

**Purpose:**

This study was designed to evaluate the effectiveness of using an SPOC-based course with a sample of nurses in terms of enhancing their knowledge and reducing their perceived barriers to sexual health communication.

**Methods:**

This prospective randomized controlled trial was conducted at a teaching hospital in northern Taiwan. One hundred fifty in-service nurses were recruited and randomly assigned to either the control group (*n* = 75), which received the standard in-service nursing education program, or the intervention group (*n* = 75), which received the SPOC-based program in sexual health communication in addition to the standard education program. The intervention group completed five 15-minute online courses followed by a 2-hr offline course. Primary outcomes (sexual health care attitudes and perceived barriers to discussing sexuality) and a secondary outcome (frequency of engaging in sexual health communication with patients) were assessed at baseline (pretest, T1) and at 1 and 3 months after module completion (T2 and T3, respectively). Generalized estimating equations were used to analyze between-group differences in pretest–posttest changes.

**Results:**

All 150 participants completed the baseline assessment; attrition occurred at the 1- and 3-month follow-ups. No participants were lost at baseline; however, follow-up attrition occurred at T2 and T3. Changes over time from baseline in attitudes toward sexual health communication and perceived barriers to discussing sexual health were significantly greater in the intervention group than in the control group. Importantly, the percentage of intervention group participants engaging in discussions with patients about sexual health increased from baseline to T2 and from T2 to T3, while no similar change over time was observed for the control group.

**Conclusions:**

The significant improvements reported for the intervention group demonstrate that cost-effective learning outcomes are achievable in spite of significant time and resource limitations. This was the first time SPOC-based learning has been used in the context of nursing education to improve sexual communication. The findings of this study suggest this format is suitable for a broader range of courses.

## Introduction

Sexual health is a fundamental component of holistic nursing care. Despite its influence on individual dignity, well-being, and quality of life, it remains a neglected topic in clinical practice. Multiple barriers, including personal beliefs, age, gender, sexual identity, and cultural taboos, hinder nurses from engaging effectively in sexual health communication (Åling et al., 2021; Annerstedt & Glasdam, 2019; Chou et al., 2023). Clinical nurses who have received training in nurse–patient communication regarding sexual health tend to be more confident in initiating related conversations (Paulsen et al., 2023; Susiladewi et al., 2025).

Although recognition of the importance of sexual health communication in nursing has been growing, access to structured and practical training remains limited. A recent systematic review of sexual health education interventions in health professional curricula revealed that, of 36 international studies, 11 targeted nursing students and only 2 targeted practicing nurses (Prize et al., 2023). In Taiwan, while national regulations mandate nurses complete 120 hr of continuing education (CEUs) every 6 years, no formalized CEU programs specifically address sexual health communication in clinical care. Effective educational interventions must enhance nurses’ competence and comfort within the constraints of heavy clinical workloads and the limited time available for continuing education. Implementing an online CEU course tailored to sexual health communication may be a flexible and accessible solution for Taiwanese nurses.

Nursing courses in sexual health care must include information on aspects of sexual health relevant to the population being served, as well as information that enables nurses to confidently engage patients in dialogue. Face-to-face workshops and simulation-based education employing problem-based learning and scenario-based strategies have been demonstrated to enhance communication skills (Kim & Shin, 2016; Lu et al., 2024). However, these methods require substantial time, human resources, and institutional support, which are often scarce in busy health care environments (Jung et al., 2023; Rezayi et al., 2022; Yang & Oh, 2023). Online courses have been shown to reduce the time and expenses needed to provide nursing education and also to be effective in improving knowledge, skills, and self-confidence (Rezayi et al., 2022).

Instruction delivered using Small Private Online Courses (SPOCs) can enhance the completion rates of continuing education units (CEUs) by limiting enrollment and enabling closer instructor–student interaction. Originating from Massive Open Online Courses, SPOCs utilize a blended learning model that integrates flipped classroom strategies with multimedia tools, making them particularly suitable for busy clinical nurses with limited time for continuing education (Atiaja & Proenza, 2016). In Taiwan, Lee et al. (2020) found nurses to be more likely to participate in CEU programs on sexual health communication when online learning was combined with face-to-face support, highlighting the importance of interactive and flexible teaching designs.

Unlike conventional online courses, which mainly focus on content delivery, the SPOC approach emphasizes a pedagogical design that integrates interactive, self-paced, and question-embedded modules to foster active engagement and critical reflection. This structure not only enhances learning motivation but also accommodates different scheduling and learning pace needs. The user-friendly interfaces and interactive features of SPOCs provide an accessible and adaptive platform that aligns with the realities of clinical practice (Baticulon et al., 2021; Chaker et al., 2024). Problem-based online learning has been shown to improve health care professionals’ knowledge (Kim & Shin, 2016) and to increase their comfort and confidence in addressing sexual health issues with patients (Areskoug Josefsson et al., 2025). Therefore, incorporating these principles into a SPOC-based CEU course is a cost-effective and sustainable strategy for strengthening nurses’ communication competence in sexual health care. Thus, it is important to evaluate whether a SPOC-based blended approach can effectively enhance nurses’ attitudes and readiness for sexual health communication.

Although the potential value of online training in sexual health communication has been highlighted in the literature, few studies have specifically examined the pedagogical effectiveness of the SPOC-based blended learning method in continuing nursing education. Therefore, this study was developed to evaluate the effectiveness of the SPOC-based teaching method in enhancing nurses’ attitudes and readiness with regard to sexual health communication. The SPOC-based learning design, characterized by interactivity, flexibility, and embedded reflective questioning, was implemented to provide an engaging and learner-centered educational experience for clinical nurses. Nurses were randomly assigned to either an intervention group, which participated in a newly developed SPOC-based blended learning program, or a control group, which received In-service nursing education program. The primary hypothesis in this study was that the intervention group would realize significantly greater improvements in sexual-health-communication-related attitudes and perceived barriers compared with the control group. The secondary hypothesis was that the intervention group would engage in sexual health discussions more frequently after the intervention than before the intervention.

## Methods

### Design

This prospective, single-blinded, two-group randomized controlled trial was implemented to evaluate the effectiveness of an SPOC-based course in improving attitudes and reducing the barriers related to sexual health communication in a sample of nurses. The trial adhered to the CONSORT (Consolidated Standards of Reporting Trials) reporting guidelines (Schulz et al., 2010) and was prospectively registered at ClinicalTrials.gov (Identifier: NCT05729672). The participants were randomly assigned to either the sexual health course (intervention) or the in-service education program (control) group. A pretest–posttest design was used to analyze the effect of the intervention on targeted outcome variables.

### Participants

Registered nurses were invited to a general nursing assembly to learn about the study. The principal investigator provided an overview of the purpose and procedures and described the inclusion criteria, participant benefits, and the right to refuse to participate. Nurses who expressed interest received detailed explanations regarding the study objectives, procedures, and ethical considerations. Inclusion criteria for nurses were (a) a registered nurse, (b) between 20 and 50 years of age, (c) Taiwan nursing ladder ≤N3 (ie, below the advanced practice/leadership rank), (d) employed full time to provide clinical nursing care, (e) in possession of a smartphone or computer with internet access; and (f) willing to participate in the study. Otherwise, eligible nurses were excluded if they were planning to resign within 3 months, unable to engage in online learning, or assigned to operating rooms, recovery rooms, palliative care units, or central supply departments. All qualified nurses who agreed to participate provided signed written informed consent.

### Randomization

The participants were assigned randomly to either the intervention or control group in a 1:1 ratio. The random sequence was generated by an independent statistician using a computer-based random number generator. Allocation concealment was ensured using sealed, opaque envelopes that were prepared in advance and opened sequentially by a research assistant after participant enrollment. To minimize potential bias, the outcome assessors and data analysts were blinded to group assignments throughout data collection and analysis. The research team members responsible for delivering the intervention were not involved in data analysis or outcome evaluation. One hundred fifty in-service nurses were enrolled as participants and randomly assigned to either the control group (*n* = 75), which received standard in-service nursing education, or the intervention group (*n* = 75), which received an additional SPOC-based course on sexual health communication.

### Sample Size Estimation

**Figure 1:**
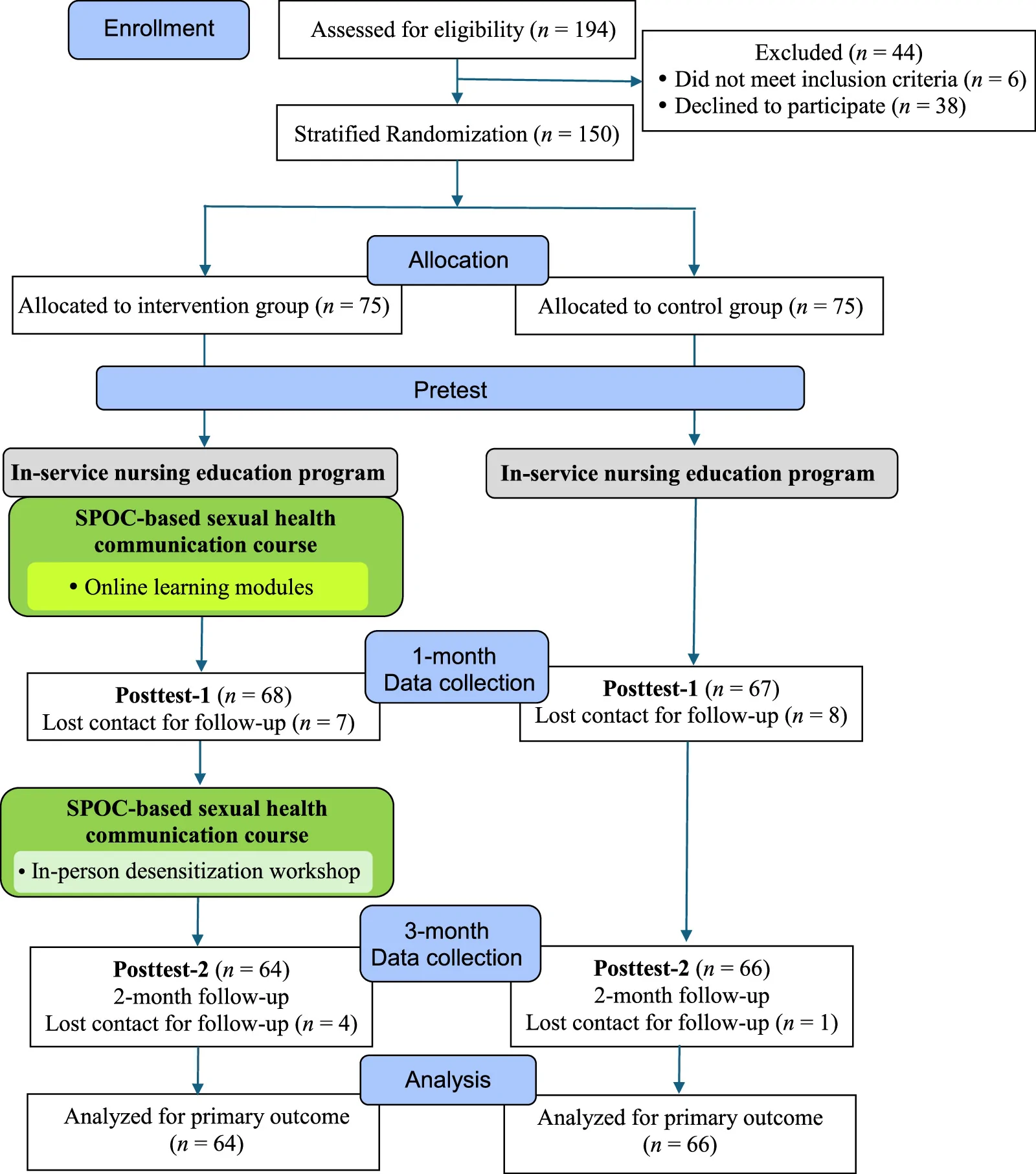
The CONSORT Flow Diagram of Participant Progress Through the Phases of the Trial *Note.* SPOC = small private online course.

Following recent studies in which similar primary outcome measures were employed, minimum sample size was calculated using G*Power 3.1.9.7 (Kim & Shin, 2016; Lu et al., 2024; Potter et al., 2025). Using a conservative medium effect (Cohen’s *d* = 0.5) for the power calculation, an alpha level of .05, and a two-tailed independent sample *t* test, a minimum of 50 participants per group was calculated. To account for an anticipated 10% attrition rate and an additional 20%–30% dropout rate during follow-up assessments, the target sample size was set at 75 participants per group, that is, 150 participants total. The primary outcome used in the analysis was the Sexual Health Care Scale–Attitudes (SHCS-A) total score. The CONSORT flow diagram (Figure 1) presents an overview of participant assignment, data collection, and analysis procedures.

### The SPOC-Based Sexual Health Communication Course

The SPOC-based Sexual Health Communication Course (“Nightingale in Gen-sEx”) was conducted in a Small Private Online Course (SPOC) format. The course was developed using the Analysis, Design, Development, Implementation, and Evaluation (ADDIE) instructional design model as the systematic framework (Dick et al., 2004). The educational design was grounded in the Transtheoretical Model (TTM) for behavior change (Prochaska & Velicer, 1997), and further informed by an interactive sexual health education framework developed by Lee and Tsai (2012). Within this framework, TTM principles are integrated to facilitate changes in attitudes and behaviors by actively engaging learners in reflective and practice-based learning activities.

The Permission, Limited Information, Specific Suggestions, and Intensive Therapy (PLISSIT) model (Annon, 1977) was adopted as the instructional framework for guiding nurse–patient communications related to sexual health, while simulation problem–based learning was used as the core teaching strategy (Kim & Shin, 2016). Internet-based multimedia videos simulating realistic clinical nurse–patient sexual health communication scenarios were developed to enhance engagement and contextual learning. Based on these pedagogical foundations, a structured curriculum was designed that encompassed learning objectives, instructional units, teaching strategies, educational activities, and content. All of the instructional materials were reviewed and validated by a panel of subject matter experts to ensure accuracy, clarity, and relevance. Following validation, the course was deployed via a smartphone-compatible and tablet-compatible Small Private Online Course (SPOC) platform. The online portion consisted of five sequential modules to be completed over a 1-month period and a subsequent 2-hr in-person desensitization workshop.

Before the intervention, participants in the intervention group (SPOC-based Sexual Health Communication Course) received a digital prelearning guide that outlined the course objectives and content and provided platform navigation instructions and login credentials. Learners were permitted to access the online platform (Taiwan Open Education Platform; https://www.openedu.tw/) at their convenience for up to 3 months. Ongoing technical support was available, and participants could post questions via the discussion forum. The framework of the SPOC-based “Nightingale in Gen-sEx” course is shown in Appendix Figure A1, Supplemental Digital Content 1, http://links.lww.com/JNR/A11.

#### Online Courses

The five 15-minute online courses included a course content area, reference materials, discussion forums, learning progress tracking, quizzes, and an instructor dashboard. Animated videos and microfilms of clinical nurse–patient communication scenarios simulated real-world interactions to enhance learner engagement and experiential learning. In three of the modules, quiz-in-video (QIV) questions (i.e., questions specifically designed to reduce passive viewing behaviors) were embedded (Habes et al., 2020). The QIVs, which appeared at specific time points, paused the program and prompted learners to answer questions before the video continued playing. If needed, the participant was allowed to review the previous portion of the video for clarification. The video resumes only when the correct response is given. The QIVs are formative learning tools and thus were not included in the course evaluation. Learner activity, including login frequency, video viewing, and quiz completion status, was automatically tracked by the platform. The participants completed a 4–5 item quiz at the end of each module that required a minimum score of 40 to advance. Unlimited attempts were permitted, with only the highest score recorded. Completion of all of the modules with passing scores was required to earn five CEU credits. Quiz scores were not used in outcome analyses, as the focus of these quizzes was on promoting engagement and course completion.

#### Offline Courses

After completing the online courses, the intervention group participants attended a 2-hr in-person workshop on sexual desensitization led by a nurse educator. This workshop aimed to reduce discomfort in discussing sexual health and strengthen communication skills. The sessions were limited to 30 participants each to ensure quality and accommodate participants’ varied schedules. Conducted in a psychologically safe setting, the workshop promoted self-exploration and interpersonal openness. The nurse educator modeled effective communication behaviors during the workshop. Key teaching strategies used included guided reflection, experiential activities, and live demonstrations, providing opportunities for participants to observe, practice, and internalize appropriate skills for sexual health discussions in clinical care.

#### Control Group

During the 3-month period of active data collection (baseline to follow-up), the participants in the control group continued participating in the in-service education programs provided by the hospital, with no other educational intervention provided. Outcome assessments were completed at the same three designated time points for both the control and intervention groups. To ensure ethical equivalence, the control group participants were granted full access to the online learning platform, including login credentials and all instructional materials, after completion of the intervention and outcome assessments.

### Measures

Baseline data were collected using structured self-report questionnaires and included participants’ demographic characteristics as well as outcome measures. Participant characteristics included age, sex assigned at birth, gender, sexual orientation, marital status, educational level, religious belief, and work-related variables. Outcome measures included attitudes regarding sexual health. Evaluation instruments included both subjective self-reported measures and objective assessments, while the process evaluation instruments focused on learner engagement with the course content and instructional design. All of the instruments were expert validated and tested for reliability prior to implementation.

#### Participant Characteristics

Data on participant characteristics, collected using a structured survey questionnaire, included age, sex assigned at birth, gender, sexual orientation, marital status, educational level, and religious belief (yes/no). Work-related information included type of hospital unit, whether their unit required nursing care related to sexual health, clinical department, professional grade, years of nursing experience, and prior exposure to sex education courses during nursing school and in the workplace. These variables were analyzed to explore potential moderating effects on learning outcomes and intervention receptivity.

#### Sexual Health Care Attitudes

Sexual health care attitudes were measured using the 17-item Sexual Health Care Scale–Attitudes (SHCS-A), originally developed by Kim et al. (2011) and adapted for nurses in Taiwan. This scale includes the four domains of discomfort in providing sexual health care, uncertainty about patients’ acceptance, concerns about colleagues’ reactions, and lack of environmental support. Each item is scored on a 3-point Likert scale, with higher total scores indicating more positive attitudes toward providing sexual health care. The internal consistency reliability of the Chinese version (C-SHCS-A) in this study was satisfactory (Cronbach α = .90). Details of the translation and cultural adaptation procedures used for the C-SHCS-A are given in Figure 1.

#### Perceived Barriers to Sexual Communication

Perceived barriers to sexual communication were assessed using a 10-item subscale adapted from an instrument developed by Hordern et al. (2009) and subsequently revised and culturally adapted for Taiwanese nurses by Chou et al. (2023). Subscale items are scored on a 5-point Likert scale (1= *strongly agree* to 5 = *strongly disagree*), with higher scores indicating greater perceived barriers. The internal consistency reliability of the subscale in this study was acceptable (Cronbach α = .79).

#### Frequency of Engaging in Sexual Health Care Communication With Patients

A single question was used to assess the secondary outcome measure on the effect of the intervention on nurse–patient sexual health care communication. The participants were asked “In the previous 30 days, have you discussed issues related to sexual health care with any of your patients? (Yes/No).” Data for this question were collected at baseline and at T2 and T3.

### Instrument Validity and Reliability

To ensure cultural relevance and applicability, all of the instruments used in this study were rigorously examined for content validity by a panel of six experts in the fields of nursing education, gender and sexuality studies, and information management. Items for each of the instruments were evaluated for relevance, clarity, and cultural appropriateness using a 3-point Likert scale (1 = *not acceptable*; 2 = *acceptable with modifications*; 3 = *acceptable*). Qualitative feedback was provided regarding suggested wording and structure for items identified as in need of modification. Revisions were made accordingly until consensus among the experts was reached. The instruments were pilot tested on a sample of 30 registered nurses to assess face validity and test–retest reliability. Two online survey rounds were administered two weeks apart, enabling item clarity, response patterns, and estimated completion time to be examined and evaluated. The results indicate the instruments have acceptable psychometric properties and are suitable for use in this study.

### Data Collection

Both groups completed the baseline survey questionnaire after enrollment and prior to randomization. The outcome data were collected at three time points: baseline (T1); 1 month postbaseline for the control group and upon completion of the online modules for the intervention group (T2); and 3 months postbaseline for the control group and 2 months after the desensitization workshop for the intervention group (T3). Identical online questionnaires requiring approximately 10–15 minutes to complete were administered at all time points. Participant recruitment occurred from October to December 2020, and follow-up assessments were completed in April 2021.

### Data Analysis

All of the received questionnaires were screened for completion, coded, and double-checked for accuracy. Statistical analyses were performed using SPSS Statistics 21.0 (IBM Corp., Armonk, NY, USA). Descriptive statistics, including frequency distributions, percentages, and means, were used to summarize participants’ sociodemographic characteristics, with between-group differences assessed using the χ^2^ test. Paired *t* tests were used in preliminary within-group comparisons (pretest vs. posttest) on the two Likert scale-scored instruments, with the significance level set at α = .05. The effectiveness of the intervention on outcome variables was further analyzed using generalized estimating equations (GEE) to analyze repeated measures both across time points (baseline, posttest, and follow-up) and between groups. Age, gender, and prior sexual health training (i.e., sex education courses received at work) were included as covariates in the GEE model to account for potential confounding effects. An exchangeable correlation structure was assumed. Behavioral changes in sexual health communication with patients were compared between groups across time points using McNemar’s test.

### Ethical Considerations

This study was reviewed and approved by the institutional review board (IRB) of the participating hospital (Approval No.: 201900190B0). Following IRB approval, the research team sought permission from the nursing department at the participating hospital to conduct institution-wide recruitment. All of the participants were fully informed of the study purpose and procedures, and of their right to withdraw at any time without penalty. Confidentiality and anonymity were strictly maintained throughout the study. All of the collected data, including demographic information, questionnaire responses, and qualitative feedback, were securely stored and accessible only to the research team. All results data were reported in aggregate form to prevent individual identification.

## Results

### Participant Characteristics

One hundred fifty registered nurses participated in this study. The mean age for the sample was 30.81 years (*SD* = 8.14; range = 21–58 years) and 82.7% were female. Most (74.7%) were unmarried and 57.3% reported having a stable partner. Most (79.3%) identified as heterosexual, with 8.7% identifying as bisexual, 8.0% as homosexual, and 4.0% as uncertain. Most of the participants (71.8%) had received sex-related education during their academic training, and 54.0% had previously attended in-service sex education programs. No statistically significant differences for any characteristics were found between the two groups. Details on the variables examined for all participants and the between-group differences are shown in Table 1.

**Table 1:** Participant Characteristics and Between-Group Differences (*N* = 150)

Characteristic	All	Intervention Group	Control Group	*p*
	(*N* = 150)	(*n* = 75)	(*n* = 75)	
	*n* (%)	*n* (%)	*n* (%)	
Age (years)				.734
≤30	96 (64.0)	47 (62.7)	49 (65.3)	
>30	54 (36.0)	28 (37.3)	26 (34.7)	
Sex assigned at birth				1.000
Male	26 (17.3)	13 (17.3)	13 (17.3)	
Female	124 (82.7)	62 (82.7)	62 (82.7)	
Gender				.919
Male	30 (20.0)	14 (18.7)	16 (21.3)	
Female	114 (75.0)	58 (77.3)	56 (74.7)	
Transgender/unsure	6 (4.0)	3 (4.0)	3 (4.0)	
Sexual orientation				.190
Heterosexual	119 (79.3)	58 (77.3)	61 (81.3)	
Homosexual	12 (8.0)	4 (5.3)	8 (10.7)	
Bisexual	13 (8.7)	8 (10.7)	5 (6.7)	
Uncertain	6 (4.0)	5 (6.7)	1 (1.3)	
Marital status				.707
Married	38 (25.3)	20 (26.7)	18 (24.0)	
Unmarried	112 (74.7)	55 (73.3)	57 (76.0)	
Partner				.322
No	64 (42.7)	35 (46.7)	29 (38.7)	
Yes	86 (57.3)	40 (53.3)	46 (61.3)	
Level of education				.315
College diploma	18 (12.0)	11 (14.7)	7 (9.3)	
University or above	132 (88.0)	64 (85.3)	68 (90.7)	
Religious belief				.740
No	62 (41.3)	32 (42.7)	30 (40.0)	
Yes	88 (58.7)	43 (57.3)	45 (60.0)	
Hospital unit				.861
General ward	86 (57.3)	42 (56.0)	44 (58.7)	
Acute and critical care unit	45 (30.0)	24 (32.0)	21 (28.0)	
Outpatient unit	19 (12.7)	9 (12.0)	10 (13.3)	
Care related to sexual health				.440
No	133 (88.7)	65 (86.7)	68 (90.7)	
Yes	17 (11.3)	10 (13.3)	7 (9.3)	
Clinical nursing rank on professional ladder				.875
N or N0	20 (13.3)	9 (12.0)	11 (14.7)	
N1	19 (12.7)	8 (10.7)	11 (14.7)	
N2	38 (25.3)	21 (28.0)	17 (22.7)	
N3	29 (19.3)	14 (18.7)	15 (20.0)	
N4 (inclusive) and above	44 (29.3)	23 (30.7)	21 (28.0)	
Sex education courses during nursing school ^a^				.959
No	42 (28.2)	21 (28.4)	21 (28.0)	
Yes	107 (71.8)	53 (71.6)	54 (72.0)	
Sex education courses at work				.413
No	69 (46.0)	32 (42.7)	37 (49.3)	
Yes	81 (54.0)	43 (57.3)	38 (50.7)	

*Note.* Clinical rank indicates level nursing on Taiwan’s nursing ladder: N or N0 = novice; N1= entry-level and requiring close supervision; N2 = competent to provide effective care with general guidance; and N3 = no supervision needed.

^a^ Missing data.

### SPOC-Based Course Effectiveness

The 64 participants who completed the five online modules and data for all three time points received passing grades and five CEU credits for completing the SPOC-based course in sexual health communication. The efficacy of providing a sexual health communication course using a SPOC-based format was analyzed by comparing changes between pretest and posttest measures for the intervention and control group. Because the primary measures for sexual health care attitudes and barriers to sexual communication were assessed using Likert scale instruments, these variables are presented separately from the stages of learning preparedness.

#### Changes in Sexual Health Care Attitudes and Barriers to Sexual Health Communication

The intervention group demonstrated significant improvements in sexual health care attitudes between pretest (T1) and T2 (*p* < .001, Cohen’s *d* = 1.22, 95% CI [0.86, 1.57]) and between T1 and T3 (*p* < .001, *d* = 1.45, 95% CI [1.07, 1.83]). Conversely, the control group showed no significant change between T1 and T2 (*p* = .272, *d* = 0.05, 95% CI [–0.28, 0.38]) and a small improvement between T2 and T3 (*p* = .006, *d* = 0.28, 95% CI [–0.06, 0.62]). Regarding barriers to sexual communication, the intervention group reported substantial reductions at T2 (*p* < .001, *d* = –0.95, 95% CI [–1.30, –0.61]) and T3 (*p* < .001, *d* = –1.18, 95% CI [–1.55, –0.81]), while the control group showed minimal change (Table 2). These large effect sizes suggest the intervention had a meaningful and sustained positive influence on the sexual health attitudes of the participants and reduced their perceived communication barriers.

**Table 2:** Pretest-Posttest Changes in Sexual Health Care Attitudes and Perceived Barriers to Sexual Health Communication in the Intervention and Control Groups

Variable/Group	Pretest	T2	*p* ^a^	Cohen’s *d* (95% CI)	T3	*p* ^b^	Cohen’s *d* (95% CI)
	Mean (*SD*)	Mean (*SD*)			Mean (*SD*)		
Sexual health care attitudes ^c^							
Intervention (*n* =75/68/59)	25.75 (6.54)	33.90 (6.88)	<.001	1.22 [0.86, 1.57]	35.20 (6.47)	<.001	1.45 [1.07, 1.83]
Control (*n* =75/67/62)	26.04 (7.13)	26.37 (7.07)	.272	0.05 [–0.28, 0.38]	28.15 (7.90)	.006	0.28 [–0.06, 0.62]
Barriers to communication ^d^							
Intervention (*n* = 75/68/59)	35.81 (8.23)	28.35 (7.33)	<.001	–0.95 [–1.30, –0.61]	26.25 (7.93)	<.001	–1.18 [–1.55, –0.81]
Control (*n* = 75/67/62)	35.75 (7.48)	35.19 (7.61)	.193	–0.07 [–0.40, 0.26]	33.37 (8.30)	.003	–0.30 [–0.64, 0.04]

*Note.* Values are presented as means (*SD*). Pretest = baseline (T1); T2 = 1 month after baseline; T3 = 3 months after baseline (i.e., 2-month follow-up).

^a^ Difference between Pretest and T2. ^b^ Difference between Pretest and T3. ^c^ Measured with the Sexual Health Care Scale–Attitudes (SHCS-A), with higher scores indicating more positive attitudes; ^d^ Measured with the Perceived Barriers to Sexual Communication scale, with lower scores indicating fewer barriers.

A GEE analysis was conducted to examine the effects of the intervention on participant attitudes toward sexual health care across the three time points. After adjusting for age, gender, and prior participation in sex education courses, the results indicate a significant time effect and significant group-by-time interaction (Table 3). The intervention group demonstrated a greater mean improvement in sexual health care attitudes at both follow-ups than the control group. These findings suggest the intervention effectively enhanced positive attitudes toward sexual health care over time. No significant between-group differences were observed at baseline (T1). With regard to perceived barriers to sexual health communication, the GEE analysis revealed significant time and group-by-time interaction effects (Table 3). The intervention group reported greater mean reductions in perceived communication barriers than the control group at T2 and T3. These results indicate the intervention contributed to reducing the perceived barriers to discussing sexual health issues over time. As with attitudes, no significant between-group differences were observed at baseline. These results indicate the SPOC-based course effectively improved the attitudes and reduced the perceived barriers of the participants.

**Table 3:** Generalized Estimating Equation Analysis of Intervention Effects on Sexual Health Care Attitudes and Perceived Barriers to Sexual Health Communication at 1- and 3-Month Follow-ups

Outcome Variable	β	*SE*	95% CI		χ^2^	*p*
			Lower	Upper		
**Sexual health care attitudes**						
Intercept	26.040	0.818	24.437	27.643	1013.641	<.001
Group effect (reference group: control)						
Intervention	−0.293	1.110	−2.468	1.881	0.070	.791
Time effect (reference: T1)						
1 month	0.573	0.632	−0.666	1.811	0.821	.365
3 months	2.138	0.805	0.560	3.717	7.050	.008
Group × time interaction (reference: T1 × control)						
1 month	7.638	1.167	5.350	9.926	42.825	<.001
3 months	7.310	1.273	4.814	9.805	32.957	<.001
**Perceived barriers to sexual communication**						
Intercept	35.813	0.944	33.963	37.664	1438.492	<.001
Group effect (reference group: control)						
Intervention	−0.067	1.276	−2.568	2.434	0.003	.958
Time effect (reference: T1)						
1 month	−0.800	0.585	−1.947	0.347	1.869	.172
3 months	−2.422	0.813	−4.016	−0.828	8.869	.003
Group × time interaction (reference: T1 × control)						
1 month	−6.466	1.137	−8.695	−4.236	32.314	<.001
3 months	−6.936	1.409	−9.698	−4.174	24.228	<.001

*Note.* Adjust: age, gender, and sex education courses at work. T1 = pretest (baseline); 1-month = T2; 3 months = T3 (i.e., 2-month follow-up).

#### Change in Frequency of Engaging in Sexual Health Care Communication With Patients

As shown in Table 4, the percentage of those reporting discussing sexual health care with patients increased significantly in the intervention group after program participation. Compared with T1, the frequency was significantly higher at both 1-month (*p* = .039) and 3-month follow-ups (*p* = .002). However, no significant within-group change was observed for frequency in the control group across the time points.

**Table 4:** Changes in Engagement in Sexual Health Communication With Patients at Each Time Point in the Intervention and Control Groups

Response	Intervention Group			Control Group		
	Pretest (*n* = 75)	T2 (*n* = 68)	T3 (*n* = 60)	Pretest (*n* = 75)	T2 (*n* = 67)	T3 (*n* = 63)
Talked about sex						
Yes	8 (10.7)	15 (22.1)	19 (31.7)	11 (14.7)	16 (23.9)	15 (23.8)
No	67 (89.3)	53 (77.9)	41 (68.3)	64 (85.3)	51 (76.1)	48 (76.2)
*p* ^a^		.039	.002		.277	.388

*Note.* Values are presented as *n* (%). Pretest = baseline (T1); T2 = 1 month after baseline; T3 = 3 months after baseline.

^a^ Compared with pretest values using the McNemar test.

## Discussion

To the best of the authors’ knowledge, the Nightingale in Gen-sEx course represents the first application of a SPOC-based blended learning approach in continuing nursing education in the realm of sexual health communication. Compared with the control group, nurses in the intervention group demonstrated significantly greater improvements in attitudes toward sexual health communication, perceived barriers to discussing sexual health, and the initiation of sexual health discussions with patients. These findings support the pedagogical effectiveness of the SPOC-based teaching approach and its feasibility as a modern and scalable strategy in nursing education.

The findings indicate that the instructional approach used in this study, which prioritizes online learning supplemented by brief in-person instruction, may be a suitable and effective educational model for enhancing nurse–patient communication. Grounded in the TTM for behavior change, the proposed course provides personalized learning resources and adaptive feedback mechanisms. Also, by incorporating stage-matched learning strategies, it allows differentiated learning pathways to be tailored to nurses’ levels of readiness, thereby promoting deeper cognitive engagement and greater motivation to adopt effective communication behaviors. These findings suggest the pedagogical design of the SPOC, beyond the instructional content itself, played an important role in facilitating changes in nurses’ attitudes and readiness for sexual health communication.

In addition, the SPOC-based course implemented in this study incorporates a two-hour in-person workshop featuring sexual desensitization and communication practice that was designed to support the internalization of positive attitudes and confidence. This workshop was designed to help compensate for the limited instructor–learner interaction typically associated with online learning and to address potential gaps in instructional guidance and scaffolding. Moreover, the face-to-face support facilitates a psychologically safe environment for skills rehearsal, emotional reflection, and peer feedback. By combining asynchronous video-based instruction with brief in-person engagement, the workshop component mitigates several common limitations of online education, including low interactivity and reduced learner motivation. Key instructional strategies, including embedded quizzes, behavioral modeling, and scenario-based learning, were integrated into the platform and aligned with evidence-based approaches shown to be effective in health education video design (Xiao et al., 2024). Collectively, these pedagogical features illustrate how an SPOC-based blended learning structure may enhance engagement, promote reflection, and facilitate the translation of learning into clinical communication practice.

The multimodal design used, which was intended to enhance nurse confidence and competence, may have alleviated the emotional and psychological discomfort associated with discussing sexual topics, thereby improving attitudes and reducing perceived barriers to sexual health communication. The significant increase in the proportion of participants in the intervention group who discussed sexual health care with patients at T2, followed by the further increase at T3, suggests a sustained effect of the SPOC-based teaching approach on clinical communication behavior. In contrast, no significant changes were observed in the control group at any time point.

Although sexuality education programs for nurses have been shown to improve knowledge and attitudes (Kim & Shin, 2016; Lu et al., 2024), these improvements have not necessarily translated into sustained behavioral changes in clinical practice (O’Connor et al., 2019; Susiladewi et al., 2025). Kumah et al. (2025) synthesized 31 studies of blended learning approaches designed to strengthen healthcare professionals’ competencies in sexual and reproductive health (SRH). Their findings revealed blended learning consistently improved knowledge, self-efficacy, and clinical performance across diverse contexts. However, most of the interventions in the included studies focused on general SRH topics such as contraception, HIV prevention, and gender-based violence, and were implemented primarily in low- and middle-income countries. Their review also highlighted the limited empirical evidence regarding structured, theory-driven blended learning models designed to promote sustained behavioral change in clinical communication.

This study builds on the existing evidence and extends the literature both by providing empirical support from a high-resource Asian context and by addressing a highly sensitive area of practice, namely sexual health communication. The findings demonstrate that an SPOC-based blended learning model grounded in the TTM can effectively enhance the attitudes and readiness of nurses to engage in sexual health discussions and facilitate the translation of attitudinal gains into observable behavioral improvements. Whereas Kumah et al. emphasized the general capacity-building potential of blended learning in SRH education, the findings of this study illustrate how a structured and theory-informed SPOC design may be used to address the emotional, cognitive, and contextual barriers that commonly hinder sexual health communication in clinical practice.

The SPOC-based blended learning framework proposed in this study, although primarily designed to enhance nurse–patient sexual health communication, may also be applicable to broader public health and community education contexts in which privacy concerns are less pronounced. For example, similar instructional principles may be cautiously adapted to enhance healthcare professionals’ gender sensitivity, communication skills related to adolescent reproductive health, and literacy regarding gender diversity and health. These issues may particularly benefit from learning approaches that are interactive, reflective, and readily accessible. By leveraging structured online modules and adaptive feedback mechanisms, SPOC-based approaches may enhance learner engagement while maintaining scalability and cost-effectiveness.

In summary, the structure and implementation of the Nightingale in Gen-sEx course provide a comprehensive and evidence-informed teaching framework for advancing the sexual health communication competencies of nurses. By combining theoretically grounded digital content with experiential learning, this SPOC-based blended learning model addresses both the cognitive and affective barriers that commonly hinder communication on sensitive topics. The demonstrated effectiveness of this approach highlights its potential as a sustainable, scalable, and pedagogically robust model for continuing nursing education in contemporary clinical settings.

### Limitations

This study was affected by several limitations. First, the data were self-reported, which may have biased participant responses and introduced social desirability bias, potentially affecting the accuracy of self-reported communication behaviors. In the future, objective or performance-based assessments (e.g., simulated patient evaluations or observational checklists) may be used to complement self-reported measures. Second, this study was conducted at a single medical center in northern Taiwan, which may limit the generalizability of the findings to other health care settings and cultural contexts. Multicenter studies across different institutions and regions are recommended to enhance external validity. Third, the relatively short follow-up period (3 months) limits understanding of the long-term sustainability of the intervention effects. Future longitudinal research with extended follow-up is warranted to examine whether the observed attitudinal and behavioral improvements persist over time.

Finally, although the control group received standard in-service nursing education, it did not include specific training in sexual health communication. This difference may have resulted in clearer between-group differences; however, it may also limit the generalizability of the findings. Future studies should consider using active control groups with comparable content to better evaluate the relative effectiveness of SPOC-based interventions.

### Conclusions

The findings of this study support that the SPOC-based “Nightingale in Gen-sEx” course has the potential to improve the sexual health communication attitudes of nurses and their perceptions regarding nurse–patient sexual health communication. This initial investigation into the efficacy of this blended learning platform, designed to provide critical instruction in sexual health communication to busy nurses, provides a replicable and adaptable blueprint for future educational efforts aimed at fostering behavioral change in resource-constrained and time-constrained clinical environments. Additional studies with larger sample sizes and follow-up studies are suggested.

## Supplementary Material
