# Supplementary material for: Effectiveness of a Small Private Online Course in Sexual Health Communication for Registered Nurses: A Randomized Controlled Trial
Source: J Nurs Res. 2026 Aug 19;34(5):e479. doi: 10.1097/jnr.0000000000000751 (PMC13608322; doi:10.1097/jnr.0000000000000751)
Supplement: Supplementary file 1 [file jnr-34-e479-s001.docx]

**Appendix Figure A1**

*The Framework of the Small Private Online Courses-Based “Nightingale in Gen-sEx” Course*
